# Relating the Structure of Noise Correlations in Macaque Primary Visual Cortex to Decoder Performance

**DOI:** 10.3389/fncom.2018.00012

**Published:** 2018-03-05

**Authors:** Or P. Mendels, Maoz Shamir

**Affiliations:** ^1^Department of Cognitive Sciences, Ben-Gurion University of the Negev, Beersheba, Israel; ^2^Zlotowski Center for Neuroscience, Ben-Gurion University of the Negev, Beersheba, Israel; ^3^Department of Physiology and Cell Biology, Faculty of Health Sciences, Ben-Gurion University of the Negev, Beersheba, Israel; ^4^Department of Physics, Faculty of Natural Sciences, Ben-Gurion University of the Negev, Beersheba, Israel

**Keywords:** population coding, population vector, optimal linear estimator, eigendecomposition, collective modes of fluctuation

## Abstract

Noise correlations in neuronal responses can have a strong influence on the information available in large populations. In addition, the structure of noise correlations may have a great impact on the utility of different algorithms to extract this information that may depend on the specific algorithm, and hence may affect our understanding of population codes in the brain. Thus, a better understanding of the structure of noise correlations and their interplay with different readout algorithms is required. Here we use eigendecomposition to investigate the structure of noise correlations in populations of about 50–100 simultaneously recorded neurons in the primary visual cortex of anesthetized monkeys, and we relate this structure to the performance of two common decoders: the population vector and the optimal linear estimator. Our analysis reveals a non-trivial correlation structure, in which the eigenvalue spectrum is composed of several distinct large eigenvalues that represent different shared modes of fluctuation extending over most of the population, and a semi-continuous tail. The largest eigenvalue represents a uniform collective mode of fluctuation. The second and third eigenvalues typically show either a clear functional (i.e., dependent on the preferred orientation of the neurons) or spatial structure (i.e., dependent on the physical position of the neurons). We find that the number of shared modes increases with the population size, being roughly 10% of that size. Furthermore, we find that the noise in each of these collective modes grows linearly with the population. This linear growth of correlated noise power can have limiting effects on the utility of averaging neuronal responses across large populations, depending on the readout. Specifically, the collective modes of fluctuation limit the accuracy of the population vector but not of the optimal linear estimator.

## Introduction

The information provided by a neuronal population depends on the relationship among several quantities: the signal provided by the population (i.e., how the mean response of each neuron varies with stimulus parameters—the tuning); the variability of the neuronal population, particularly the structure of variability that is shared between neurons (often termed “noise” correlations); and the manner in which the information is extracted (the decoder and its weights) (Georgopoulos et al., 1982; Salinas and Abbott, 1994; Abbott and Dayan, 1999; Deneve et al., 1999; Sompolinsky et al., 2001; Averbeck et al., 2006; Shamir, 2006; Shamir and Sompolinsky, 2006; Ecker et al., 2011; Graf et al., 2011).

Numerous studies have described the structure of shared fluctuations in neuronal populations, reporting that noise correlations (hereafter referred to as simply “correlations”) depend on the physical and functional distance between neurons (Smith and Kohn, 2008; Rothschild et al., 2010; Cohen and Kohn, 2011; Smith and Sommer, 2013) as well as other features that may lead to shared variability (Cui et al., 2016). More recent work has emphasized that correlations arise from a low-dimensional form of dependency—in the simplest scenario, correlations may arise from a single fluctuation shared throughout the population (Ecker et al., 2014; Lin et al., 2015; Schölvinck et al., 2015, but see Rabinowitz et al., 2015; Rosenbaum et al., 2017).

Previous work that has investigated the effect of correlated variability on information has usually compared information in raw and shuffled (randomly permuting across trials to strongly reduce correlations) data (Gawne and Richmond, 1993; Panzeri et al., 1999; Petersen et al., 2002; Romo et al., 2003; Averbeck and Lee, 2006; Graf et al., 2011). This provides a quantitative evaluation of how shared variability affects information. However it does not provide much insight into the outcome—how much of the effect should be attributed to the distribution of noise? How much to the distribution of the signal? How much depends on the choice of readout algorithm? The importance of the relationship between these three factors is made clear by recent progress in understanding which correlations limit information in large neuronal populations (Moreno-Bote et al., 2014; Kanitscheider et al., 2015; Kohn et al., 2016)—those termed differential correlations. Differential correlations limit the performance of any linear estimator (but not that of estimators that can extract information from the higher order statistics of the neuronal responses Shamir and Sompolinsky, 2002, 2004) because they introduce fluctuations in the same direction as the signal that is being extracted.

Here we apply new methods—developed in previous theoretical work (Shamir, 2014) but not previously applied to physiological data—to analyse the structure of correlations in populations of V1 neurons. All data analyzed here is courtesy of the laboratory of Adam Kohn. Specifically, we conduct an eigendecomposition of the correlation matrix. Consistent with previous work, we find that much of the observed correlated variability involves a uniform mode fluctuation mode, although there is additional significant structure as well. We use the eigendecomposition to clarify how two common decoders—population vector and optimal linear estimator (OLE)—are affected by structured variability. Both the population vector and the OLE are linear readout mechanisms. The population vector (Georgopoulos et al., 1982), does not take neuronal noise correlations into account and modeling studies predicted that its accuracy will be limited due to the correlated noise (Sompolinsky et al., 2001). By construction, the accuracy of the OLE is superior to that of the population vector, but the OLE requires some degree of fine-tuning (Shamir and Sompolinsky, 2006). This has led people who believe in optimality principle of computation in the brain to reject the population vector as a valid hypothesis for the neural code. However, the question of the neural code is a scientific question that should be addressed experimentally and not based upon belief. For instance, the reduction of correlations during attention (Cohen and Maunsell, 2009) is not easy to reconcile with the expected performance of the OLE. Here, we show how the differences between the performance of these decoders on raw and shuffled data can be understood by examining the relationship between the decoder weights and the eigenvectors of the correlation matrix.

## Materials and methods

All measures of variability are ±1 std, unless otherwise noted.

### Experimental procedures

All data analyzed here is courtesy of the laboratory of Adam Kohn. Experimental procedures have already been reported in the past and described in detail (Smith and Kohn, 2008). Briefly, neural activity was recorded using the “Utah” Array from the primary visual cortex of anesthetized monkeys (macaca fascicularis) while the monkeys were presented with visual stimuli. The array consists of a 10 × 10 grid of microelectrodes spaced 400 μm apart.

The visual stimuli were oriented drifting gratings presented in a circular aperture surrounded by a gray field of average luminance (8 orientations in 5 datasets and 36 orientations in 3 datasets). Stimuli were presented binocularly, for 300–400 ms, and separated by 500–800 ms intervals during which we presented an isoluminant gray screen. Each stimulus was presented 200–400 times. For each experiment (data set) stimulus presentation times, inter-stimulus duration and the number of trials per stimulus were fixed.

### Analysis

Let us denote the spike count of the *i*-th neuron in a population of *N* neurons to the *t*-th presentation of a grating stimulus with orientation θ during the entire duration of stimulus presentation, by ${\left\{r_{i,t}\right\}}_{i\;=\;1}^{N}$.

#### Rate tuning

For the calculation of the tuning curves, *E*[*r*_*i*_|θ], the firing rate in each trial was calculated using a time window from stimulus onset to stimulus offset. The tuning curves were then fitted using the Von-Mises function: $A_{i}e^{k_{i}\cos\left[2\left(\theta -\varphi_{i}\right)\right]}$, where θ is the stimulus orientation and φ_*i*_ is the preferred orientation of the cell. As we are interested in the functional dependence of the correlations, i.e., in the dependence on the preferred orientation, and in comparing the population vector to the OLE, we discarded from the analysis cells that did not show a good fit. This is because the preferred orientation of a cell with poor tuning is meaningless. The goodness of fit was defined as one minus the mean square of the deviation of the tuning curve (i.e., mean firing rate for a given stimulus) from the Von-Mises fit over the variance of the tuning curve, $Goodness=1-\frac{\underset{\theta }{\sum }{\left(E\left[r_{i}|\theta \right]-f_{i}\left(\theta \right)\right)}^{2}}{\underset{\theta }{\sum }{\left(E\left[r_{i}|\theta \right]-E\left[r_{i}\right]\right)}^{2}}$, and a threshold of 0.5 was used except for **Figure 3** where the spatial and not the functional structure was of interest. In all, there were 675 units out of which 457 met our goodness of fit criterion. The exact value of threshold of 0.5 for the goodness of fit did not change qualitatively our results. This should not be taken to imply that cells with poor tuning cannot contribute to the information content of the population response, see e.g., Zylberberg (in review).

#### Correlations

The trial-to-trial fluctuations of the neural response from its conditional mean, given the stimulus, δ*r*_*i,t*_ = *r*_*i,t*_ − *E*[*r*_*i*_|θ] yield the noise that limits the accuracy of a linear readout. Due to this reason the correlation coefficients that are presented here are the correlation coefficients of these deviations from the mean firing.

For shuffled data the distribution of correlation coefficients was well fitted (not shown) by Gaussian distribution with zero mean and variance of $1/\sqrt{\text{trials per stimulus }\times \text{ number of stimuli}}$. Shuffling was done by randomly permuting all trials for each neuron given a specific stimulus.

**Figure 1:**
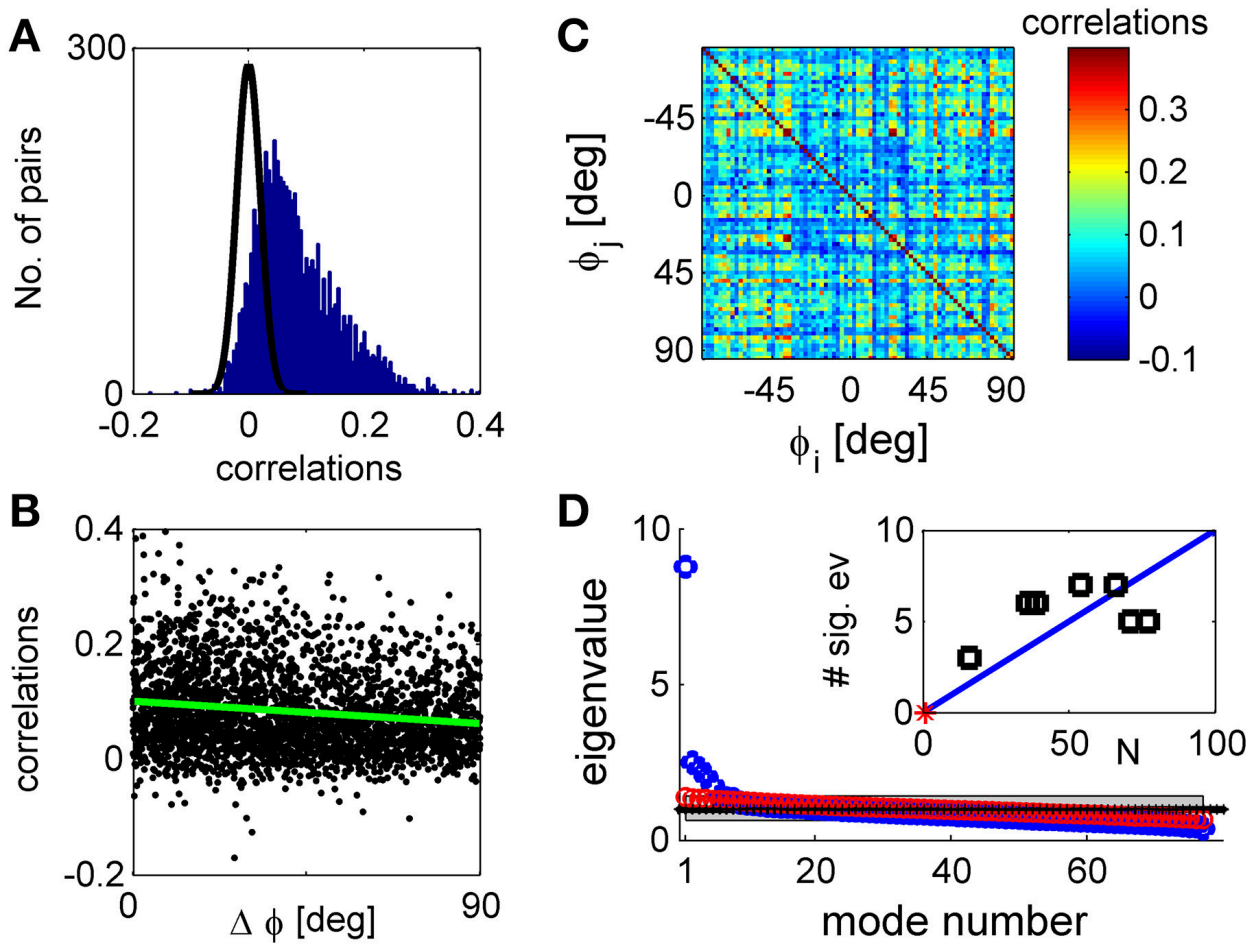
Noise correlations and eigenvalue spectrum. **(A)** The histogram of correlation coefficients of the response fluctuations of pairs of 77 simultaneously recorded orientation tuned neurons (blue histogram). The black line shows a Gaussian distribution with zero mean and standard deviation of $1/\sqrt{\text{trials per stimulus }\times \text{ number of stimuli}}$ that very well approximates the distribution of correlation coefficients in shuffled data. **(B)** The functional dependence of the correlations. The pair-wise correlation coefficients are plotted as a function of the preferred orientation difference of each pair. The green line depicts a linear fit of the functional dependence with a slope of −4e-4 deg^−1^ with standard error of 5e-5 deg^−1^. **(C)** The correlation matrix shown in color code as function of the neuron's preferred orientation. **(D)** The eigenvalues of the correlation matrix are shown by rank order (blue circles). For comparison, the eigenvalues of a typical correlation matrix of shuffled data are also shown (red circles). The gray region depicts the range between the maximal eigenvalue obtained over 1,000 realizations of shuffled data and the minimal one. In the limit of infinite number of trials all eigenvalues of shuffled data will be one (black dotted line). The inset shows the number of significant eigenvalues in the different datasets as function of the number of tuned units in each dataset. The red asterisk shows the point of zero significant eigenvalues for population of *N* = 1 neurons. The solid blue line shows a linear regression fit through (1,0) with a slope of 10%, *p* < 0.001 *t*-test (*n* = 6).

**Figure 2:**
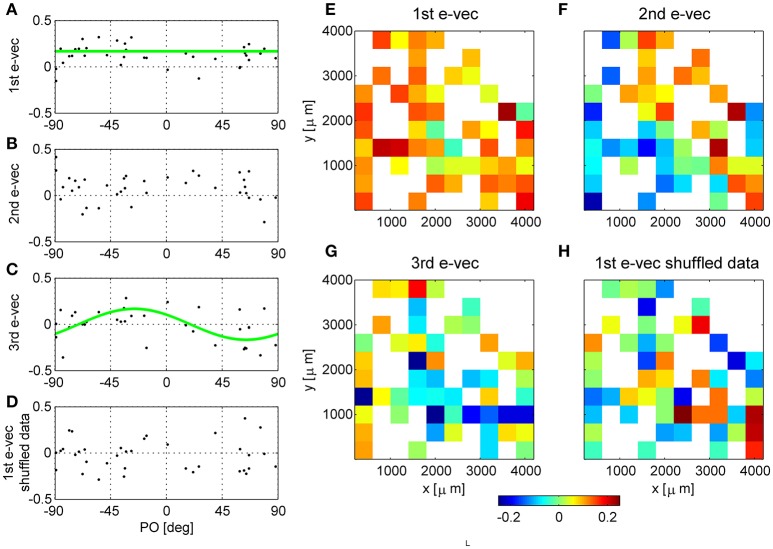
Functional and spatial structure of the collective modes of fluctuation. **(A–D)** Present functional structure of modes in one dataset. **(E–H)** Show the spatial structure of modes in another dataset. The eigenvectors corresponding to the first **(A)**, second **(B)**, third **(C)** eigenvalues and shuffled data **(D)** are shown in the functional space representation, i.e., element k of the vector is shown as function of the preferred orientation of neuron k. The green line in **(A)** depicts the mean of the eigenvector. The green line in **(C)** shows a cosine fit. The squared projection of the first eigenvector onto the uniform direction is (**v**^(1)^ · **u**)^2^ = 0.56, whereas, for comparison the projection of a random direction is expected to be zero on average with variance of 1/*N* = 1/36 = 0.028 in this example. **(E–H)** Show the first, second, third eigenvectors of raw data, and the principle eigenvector of shuffled data (respectively) in their spatial representation. The matrices represent the location of the electrode (ten by ten Utah array) and the color depicts the value of the eigenvector at that entry. In case several neurons were separated from the same electrode we present their mean.

**Figure 3:**
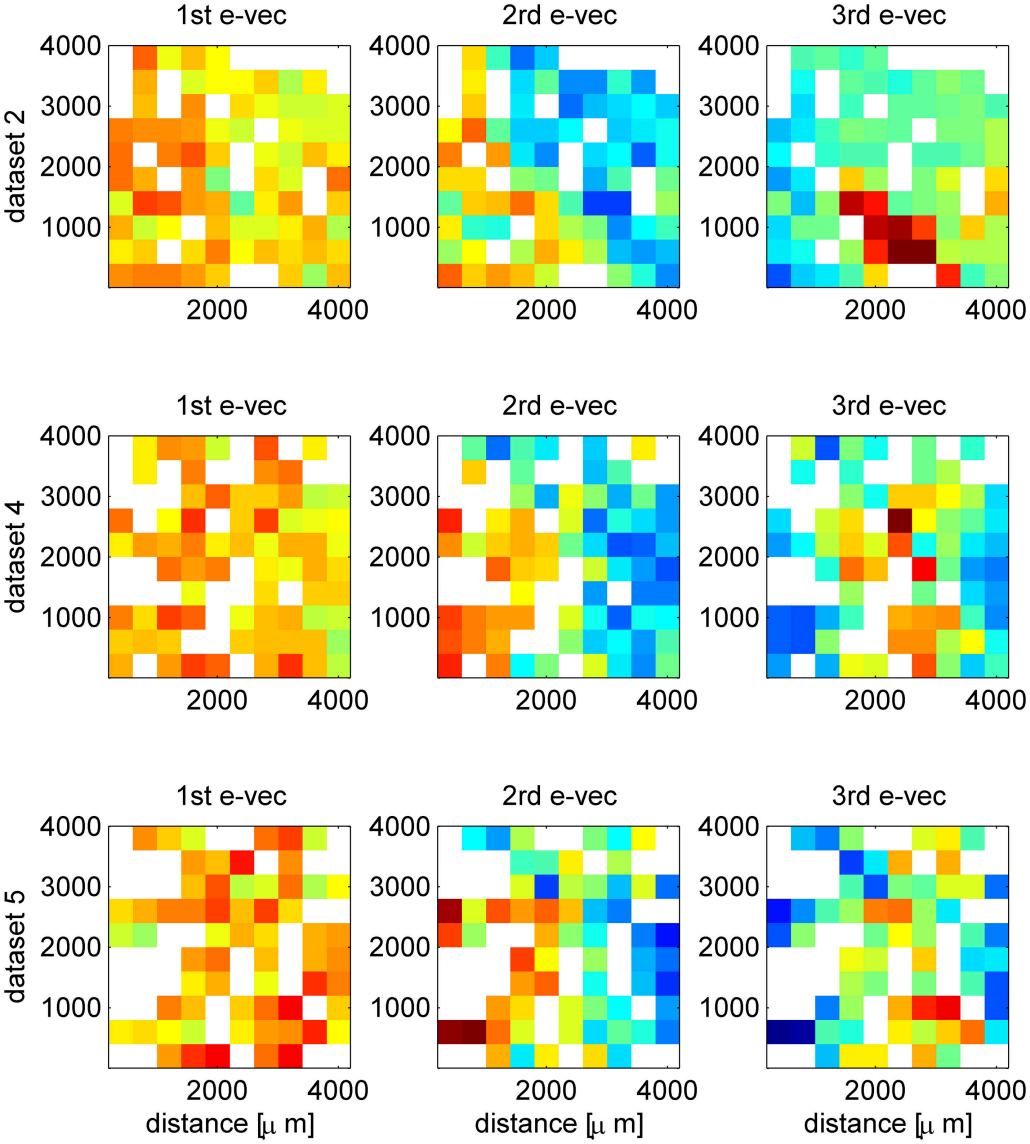
Additional examples of spatial structure of eigenvectors. The first, second and third eigenvector are shown (by column) in their spatial representation, for three simultaneously recorded data sets (rows). The matrices represent the location of the electrode (ten by ten Utah array) and the color depicts the value of the eigenvector at that entry. In case several neurons were separated from the same electrode we present their mean. The eigenvectors were computed from the correlation matrix of the inter-stimulus interval data, which provides a much clearer structure. Here, to obtain a less noisy display we used also units that showed poor orientation tuning, as we are interested in the spatial structure and not the functional. To appreciate the stability of the eigenvector structure one may compare the first row here (dataset 2 with all *N* = 129 single units) and Figures 2E-G that show the results for the same dataset but with all *N* = 71 *tuned* single units. The picture that emerges from this figure is of a hierarchy of spatial structures. Starting with a uniform mode and showing increasingly finer structure for higher order modes.

**Figure 4:**
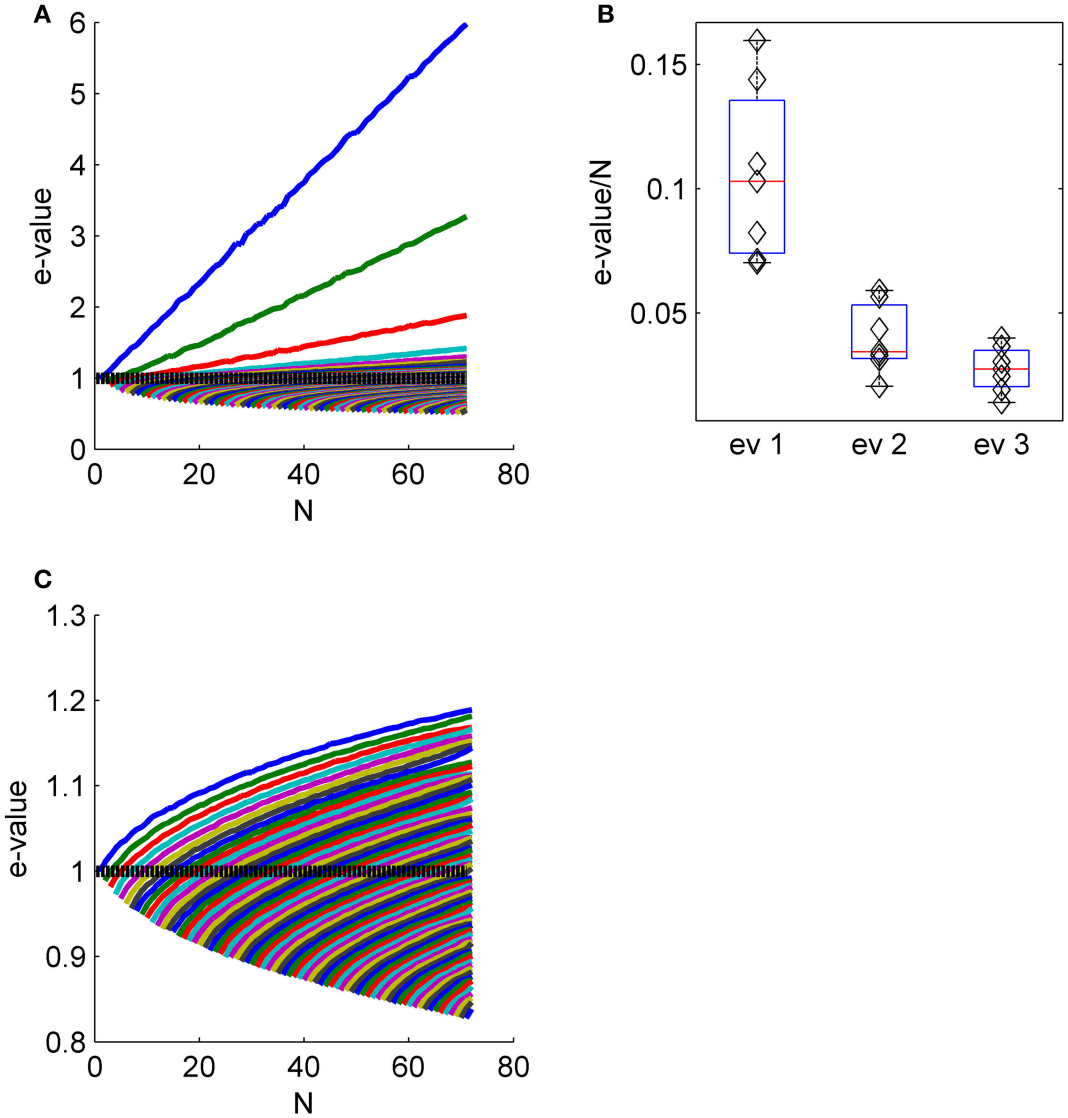
The collective nature of eigenvalues. **(A)** The eigenvalue spectrum of the noise correlation matrix is shown as function of the population size (same data set as in Figures 2E-G). The dashed line shows the spectrum for independent neural population, that is the shuffled data in the limit of infinitely many trials. **(B)** The distribution of the slope of the linear scaling of the first, second and third eigenvalues with the population size across the different data sets. **(C)** The eigenvalue spectrum as in **(A)** for shuffled data. Note the different scale of **(A,C)**.

The analysis of the correlation matrix structure (e.g., Figures 1–**4**) was done using correlation matrices that were averaged over all stimulus conditions (i.e., grating orientation). Consequently, the results we have shown are for “stimulus independent” correlations. We confirmed that the correlation structure was similar for each grating orientation separately as well as for spontaneous activity measured during the interstimulus interval, consistent with previous findings (Kohn and Smith, 2005).

One dataset (dataset 2, shown in Figures 2E–H, 3, top row, Figure 4) showed some measure of non-stationarity around the start and the end of the experiment. To prevent the non-stationarity from affecting our results we excluded the first 59 and last 6 trials from our analysis. Interestingly, restricting trials did not change any qualitative result. Moreover, the structure of the principle eigenvectors remained extremely stable (even when including non-stationary trials) and the main quantitative effect was in the eigenvalues themselves.

#### Significance of eigenvalues

In the limit of large (number of trials/neuron) the eigenvalues in the shuffled data will have a finite support (given by Marchenko-Pastur distribution), and there will be zero probability of finding eigenvalues beyond that specific interval around one. As the number of trials in our dataset is considerable, the overwhelming majority of eigenvalues of the shuffled data fall within the bounds of the Marchenko-Pastur distribution, and those that fall beyond are extremely close. To reflect the nature of the eigenvalue distribution (of the shuffled data) we defined an eigenvalue to be significant if it was larger than the maximal eigenvalue in 1,000 realizations of shuffled data.

#### Population vector and optimal linear readout

**Figure 5:**
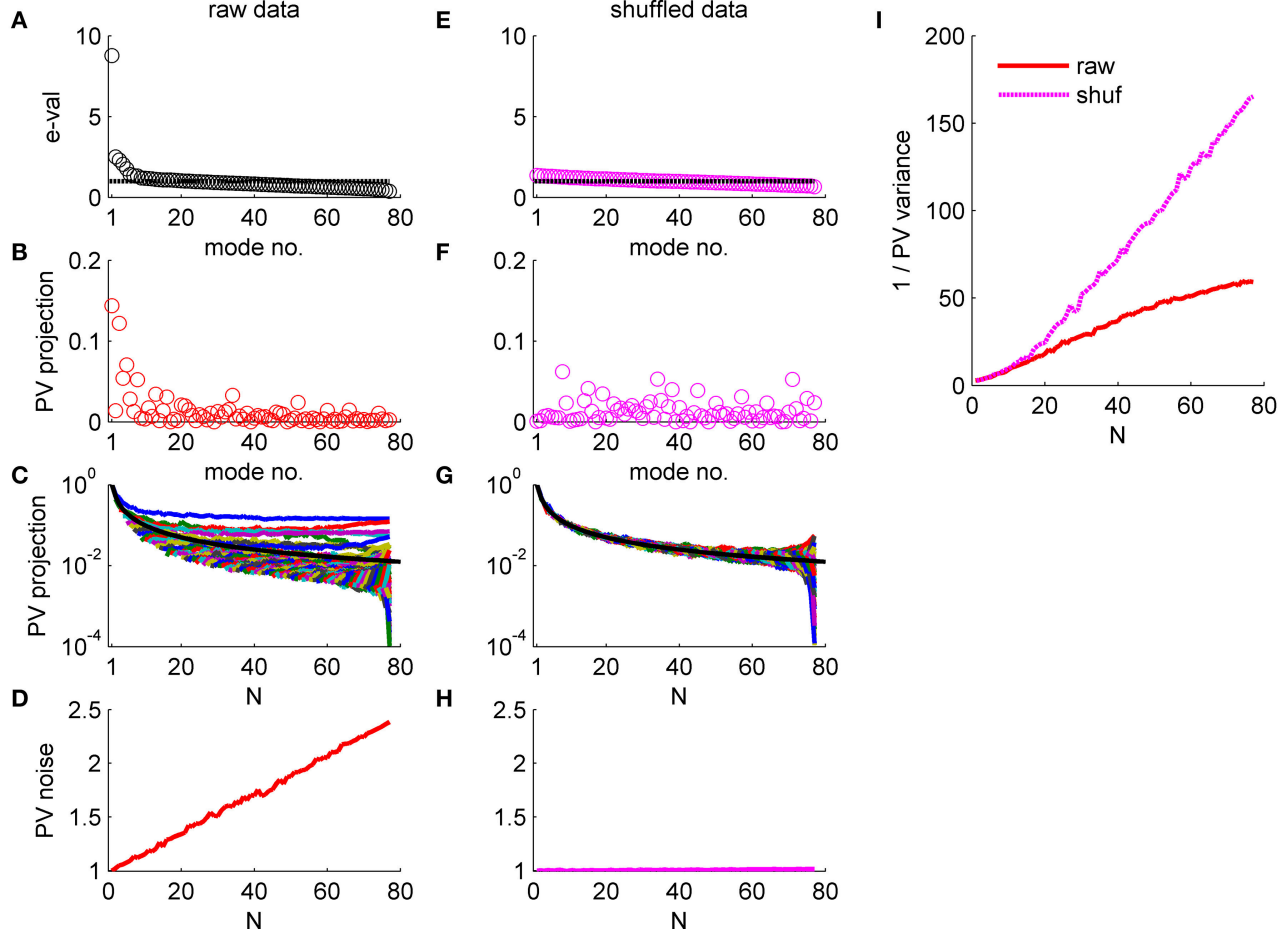
Population vector accuracy in a correlated heterogeneous population, example 1. **(A)** Noise distribution. The eigenvalue spectrum of the noise-correlation matrix is presented by rank order. Dashed line shows the spectrum in the uncorrelated case. **(B)** The squared overlap between the population vector weights **ff2**$=\left(e^{i2\varphi_{1}},e^{i2\varphi_{2}},\ldots e^{i2\varphi_{N}}\right)/\sqrt{N}$ and the eigenvectors of the correlation matrix, arranged by their eigenvalue rank order. **(C)** The squared overlap between the population vector as function of the population size. The overlap with different eigenvectors is depicted by color. The black line shows for comparison the expected overlap of two *N*-dimensional random vectors, which is *1/N*. **(D)** Population vector noise. The total variability of the population vector subspace, which is the sum of the noise distribution in **(A)** weighted by the projection in **(B)**, is shown as function of the population size. **(E-H)** Same plots as in **(A-D)**, respectively, for a single iteration of shuffled data. **(I)** Population vector angular accuracy. One over the variance of the population vector angular estimation error is shown as function of the population size for the actual and shuffled data in solid red and dashed magenta, respectively.

**Figure 6:**
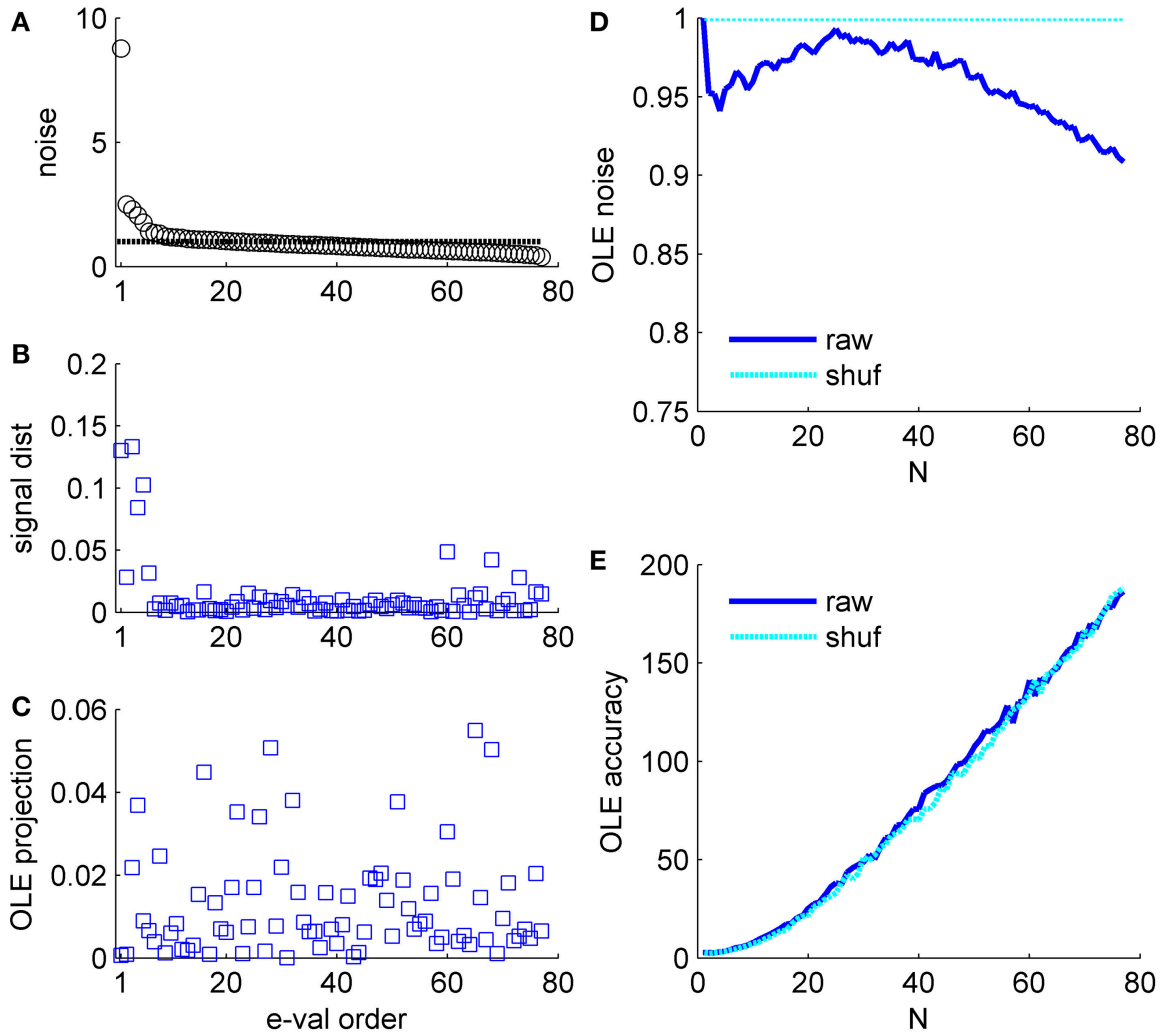
Optimal linear estimator accuracy in a correlated heterogeneous population, example 2. **(A)** Noise distribution. The eigenvalue spectrum of the noise-correlation matrix is presented by rank order. Dashed line shows the spectrum in the uncorrelated case. **(B)** Signal distribution. The squared overlap between the signal, in terms of the covariance between stimulus and neural responses (see section Materials and Methods) and the eigenvectors of the correlation matrix is shown by the eigenvalue rank order. **(C)** The squared overlap between the optimal linear estimator weights and the eigenvectors of the correlation matrix, arranged by their eigenvalue rank order. **(D)** Optimal linear estimator noise. The total variability of the optimal linear estimator, which is the sum of the noise distribution in **(A)** weighted by the projection in **(C)**, is shown as a function of the population size (blue). Cyan line shows the uncorrelated case, for comparison. **(E)** Optimal linear estimator angular accuracy. One over the variance of the angular estimation error is shown as function of the population size for the raw and shuffled data in solid blue and dashed cyan, respectively.

**Figure 7:**
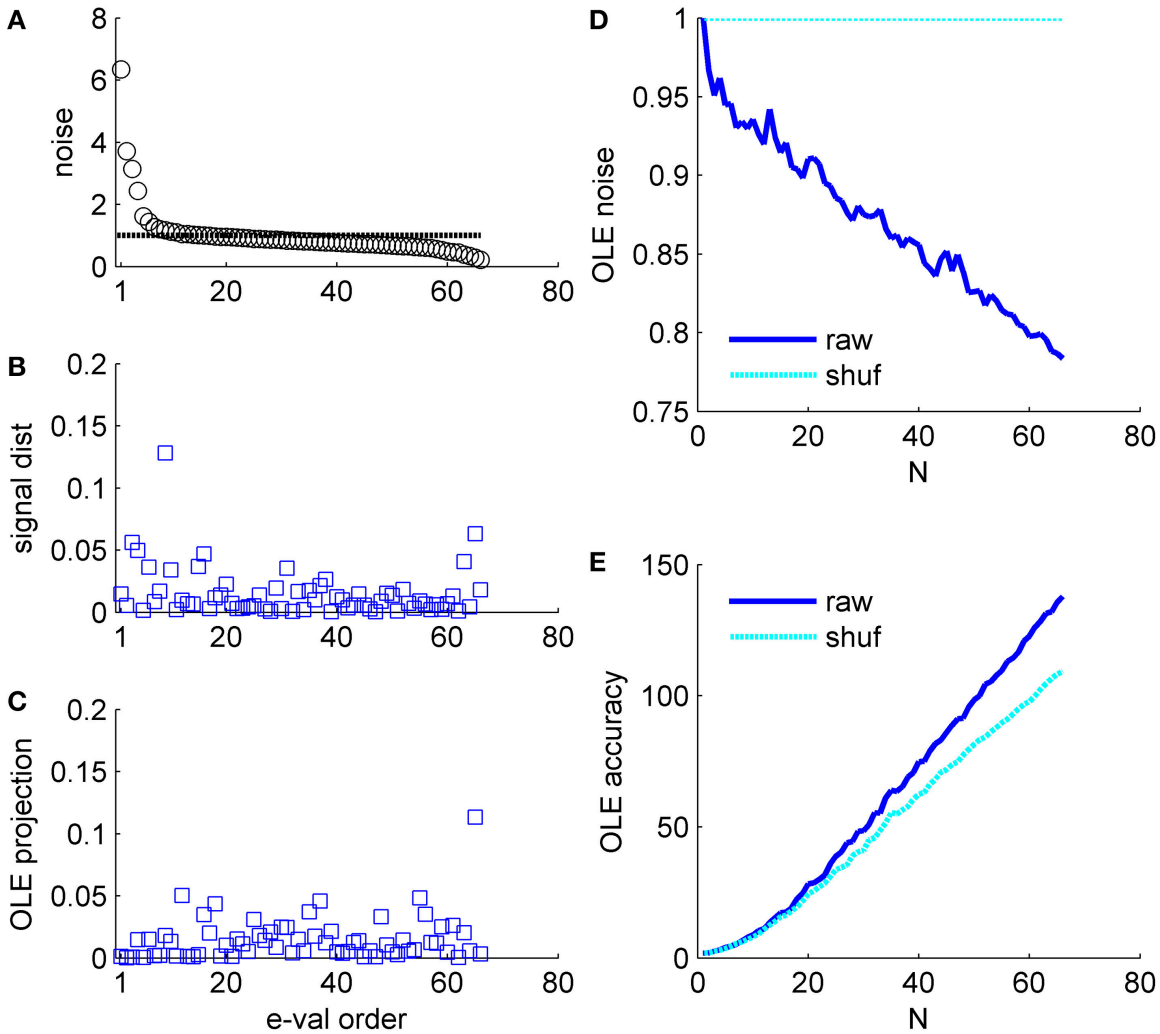
Optimal linear estimator accuracy in a correlated heterogeneous population, example 3. **(A)** Noise distribution. The eigenvalue spectrum of the noise-correlation matrix is presented by rank order. Dashed line shows the spectrum in the uncorrelated case. **(B)** Signal distribution. The squared overlap between the signal, in terms of the covariance between stimulus and neural responses (see section Materials and Methods) and the eigenvectors of the correlation matrix is shown by the eigenvalue rank order. **(C)** The squared overlap between the optimal linear estimator weights and the eigenvectors of the correlation matrix, arranged by their eigenvalue rank order. **(D)** Optimal linear estimator noise. The total variability of the optimal linear estimator, which is the sum of the noise distribution in **(A)** Weighted by the projection in **(C)**, is shown as a function of the population size (blue). Cyan line shows the uncorrelated case, for comparison. **(E)** Optimal linear estimator angular accuracy. One over the variance of the angular estimation error is shown as function of the population size for the raw and shuffled data in solid blue and dashed cyan, respectively.

In all of our calculations of the population vector and optimal linear estimator accuracy we have used half of the trials (chosen randomly with equal probability without repetitions for each stimulus condition) as training set to define the preferred orientations of the neurons and the optimal linear readout weights. The accuracy was then estimated using the rest of the trials as generalization set. For every value of population size, *n* (out of *N* in the dataset) readout accuracy (Figures 5I, 6E, 7E) was averaged over 100 repetitions of randomly choosing the training set and of choosing the subpopulation of *n* neurons out of *N* (when applies). Note that the mean squared error of each readout includes both a bias term and noise term (variance). However, as the bias can be overcome by a simple deterministic mapping and decays to zero rapidly with the population size, we focused here on the noise term.

In Figures 5-7 we present the (squared) projection of the readout weight vector on different directions. The projection of a normalized vector **x** on a normalized eigenvector **v** is simply $\frac{\text{x}\cdot \text{v}}{\left||\text{x}||||\text{v}|\right|}$ (which also defines the cosine of the angle between the two vectors). The noise of a linear readout *ẑ* = **w** · **r** as displayed in Figures 5–7D is given by **w**^*t*^**Cw**, where **C** is the correlation matrix and **x**^*t*^ denotes the transpose of **x**. The weights of the OLE can be written in a signal to noise like expression, ${\text{w}}_{\text{ole}}={\text{Q}}^{-1}\text{U}$, where the “noise” is embedded in the neuronal correlations, *Q*_*ij*_ = *E*[*r*_*i*_*r*_*j*_]. The signal for the optimal linear estimator is given by the co-variation of the signal and the neuronal responses $u_{i}=E\left[E\left[r_{i}|\theta \right]e^{i2\theta }\right]$, where *e*^*i*2θ^ is a unit vector (in the complex plane) in the direction of the stimulus, θ (the factor 2 is due to the fact that orientation has a 180° symmetry and not 360°). Signal distribution in Figures 6B, 7B shows the (squared) projection of the signal onto the (rank ordered) eigenvalues of the correlation matrix.

## Results

We analyzed 8 data sets recorded in V1 of 6 anesthetized monkeys. The neuronal populations consisted of 23–129 putative single units that we shall refer to as units or single units hereafter. Neurons were driven by drifting sinusoidal gratings of 8–36 different orientations. One data set used random phases for the stimulus. Consequently, all of its evoked data were excluded and we only present its inter-stimulus data in Figure 3. As we were interested in part in investigating the correlation structure with respect to the preferred orientation space we excluded (except for Figure 3) units that did not show a clear preferred orientation, resulting in 7 data sets of 16–77 (51 ± 22) units.

### Neuronal noise correlations are highly diverse

Consistent with many previous studies, trial-to-trial variability was correlated between neurons (Cohen and Kohn, 2011). For the dataset illustrated in Figure 1 the mean correlation was 0.07 with standard deviation of 0.07 (standard error of the mean correlation was 0.001). Most pairs had positive correlations that fell outside the distribution of correlations produced by the same responses after shuffling (randomly permuting the trials of each neuron, shown in the black line on Figure 1A). Across datasets 78 ± 7% of the pairs exhibit correlations that deviate by more than two standard deviations from the shuffled distribution mean. Correlations were stronger between pairs of neurons with similar orientation preferences than between neurons with different preferences (Figure 1B), as shown in previous work (Zohary et al., 1994; Lee et al., 1998; Smith and Kohn, 2008; Cohen and Maunsell, 2009; Rothschild et al., 2010; Cohen and Kohn, 2011; Smith and Sommer, 2013). This is demonstrated by the linear regression, green line, with a slope of−4e-4 deg^−1^ with standard error of 5e-5 deg^−1^ (that yields a decrease of about 40% of the mean correlations over 90° difference), which is significantly different than zero, *p* < 10^−3^, *t*-test (*n* = 2,924). However, correlations show considerable variability around the systematic functional dependence. This variability is even more apparent in viewing the correlation matrix (Figure 1C), in which the neurons were ordered according to their preferred orientation. This substantial diversity complicates the study of correlation structure.

### Eigendecomposition

To investigate the structure of the correlation matrix it is useful to examine its eigenvalue spectrum and corresponding eigenvectors. The utility of this approach is that, instead of studying *N(N-1)/2* pairs, the decomposition to the eigenvectors of the correlation matrix allows us to represent the fluctuations in the population response as sum of *N* modes of fluctuation that are uncorrelated with each other, where *N* is the number of neurons in the population. Each mode is characterized by its eigenvalue and its corresponding eigenvector, **v**^(*n*)^. The trial-to-trial fluctuations can then be represented as a sum of uncorrelated modes. Namely, the deviation of neuron *i* from its mean response, δ*r*_*i*_ ≡ *r*_*i*_ − *E*[*r*_*i*_|θ](see also section Materials and Methods) can be written as $\delta r_{i}=\sum_{n\;=\;1}^{N}z_{n}v_{i}^{\left(n\right)}$, where {*z*_*n*_} is a set of uncorrelated trial-to-trial fluctuations with zero mean and variance *c*_*n*_–for the raw responses these are the modes of the covariance matrix and for normalized responses (divided by the standard deviation) the correlation coefficients matrix. In addition, the eigenvalue spectrum ranks the modes by the strength of their fluctuations allowing us to focus on the most dominant modes. It is also common to refer to the eigenvectors of the correlation matrix with the largest eigenvalues as principal components.

Figure 1D shows the eigenvalue spectrum of the correlation matrix for one example data set (blue circles). For comparison, the spectrum of the correlation matrix after shuffling is shown in red circles. With unlimited data the correlation matrix of shuffled data will be a unity matrix of size *N*, and its spectrum will be flat with all eigenvalues equal to one (dashed black line). As data are finite (we used the same number of trials in the shuffled data as in the real data), the eigenvalues of the shuffled data correlation matrix will be distributed around one. The gray region depicts the eigenvalue range estimated by 1,000 realizations of the shuffled data. We defined an eigenvalue of the correlation matrix to be significant if it is larger than the maximal eigenvalue achieved by 1,000 realizations of shuffled data (see section Materials and Methods).

As can be seen from the figure, the eigenvalue distribution of the real correlation matrix is composed of a few large, significant eigenvalues and a semi-continuous tail of many eigenvalues that are small and not significantly different from the shuffled distribution. The number of significant eigenvalues varies across datasets and roughly scales linearly with the number of neurons, Figure 1D inset. On average across datasets the percentage of significant eigenvalues was 12.5 ± 5% of the population size.

### The structure of the collective modes

The eigenvectors with largest eigenvalues represent shared modes of fluctuations involving a finite fraction of the entire neural population. Figures 2A-C show the eigenvectors of the three most dominant modes in one dataset, i.e., with the largest eigenvalues. The eigenvectors are represented here as a function of the preferred orientation of each neuron. The first eigenvector (Figure 2A) involves almost all neurons (except for a few with values close to zero). A prominent feature of the first mode is that almost all of its components are of the same sign, and those with a different sign are small in absolute value. Consequently, this mode reflects a *uniform* shared mode of fluctuation in which most of the neurons increase or decrease their firing rate together. The squared projection of the first eigenvector onto the uniform direction is (**v**^(1)^ · **u**)^2^ = 0.56, where $\text{u}=\left(1,1,1\ldots \right)/\sqrt{N}$ is the uniform vector. The hallmark of this fluctuation can also be seen in the distribution of correlation coefficient that is shifted toward the positive side (see e.g., Figure 1B). We find that in all datasets the first eigenvector has a considerable overlap with the uniform direction (0.67 ± 0.15). In contrast, for shuffled data any eigenvector will be a random direction, its scalar product with the uniform direction (or any pre-determined direction) will be zero on average with variance 1/*N*.

In this example dataset, the second eigenvector (Figure 2B) also shows a significant projection on the uniform direction of (**v**^(1)^ · **u**)^2^ = 0.24, which is significantly different than zero, *p* < 0.001 (compared with a random direction). However, on average, the second eigenvector had almost no overlap with the uniform direction (0.09 ± 0.13), and neither did the third (0.035 ± 0.04).

The third collective mode shows a prominent functional structure (Figure 2C). The elements of the eigenvector with negative preferred orientation tended to be positive whereas elements with negative preferred orientation negative. This represents a mode of fluctuations in which neurons with positive preferred orientations fluctuate in an opposite manner to neurons with negative preferred orientations. Thus, neurons with similar preferred orientations will tend to fluctuate together. This mode gives rise to structure resembling a sine function. To quantify the similarity of this eigenvector to sine and cosine tuning we studied its squared projection onto the second Fourier space |**v**^(3)^ · **ff2**|^2^, where $ff2=(e^{i2\varphi_{1}},e^{i2\varphi_{2}},\ldots e^{i2\varphi_{N}})/\sqrt{N}$. In the example of Figure 2C the squared projection of the third eigenvector onto the second Fourier component was 0.12, *p* < 0.05 (compare with the projection of a random direction that is zero on average with variance of 1/*N* = 0.028). For this dataset the squared projection of the second eigenvector onto the second Fourier component was 0.006.

For comparison, we show in Figure 2D the first eigenvector of the correlation matrix of shuffled data. In the shuffled case the eigenvectors lack functional structure and their projections onto the uniform direction and onto the second Fourier subspaces are zero, on average, with variance of 1/*N*.

In some cases we found that the structure of the second and third eigenvectors was spatial. As the neurons are recorded by an array of electrodes arranged on a square lattice (400 μm spacing), we can represent the eigenvectors by the electrode location of each neuron instead of its preferred orientation. In cases where several neurons were isolated on the same electrode, we show their average. Figures 2E-G show an example of eigenvectors from a different dataset that exhibit spatial structure. The eigenvector of the first mode, as above, represents the uniform mode, Figure 2E. This is to be expected as the uniform vector will remain uniform in any representation. Examining the second eigenvector reveals a collective mode of fluctuations, in which neurons in the upper right fluctuate in anti-phase to neurons in the lower left, Figure 2F. The third mode demonstrates a collective mode of fluctuation with a finer spatial structure, Figure 2G. A clearer picture of the eigenvector structure emerges when the inter-stimulus-interval correlation matrix is used, Figure 3 (top row, note that here we used also untuned units). For comparison, Figure 2H shows the spatial structure of the first eigenvector of the correlation matrix of shuffled data. Additional examples of spatial modes from additional datasets are shown in the second and third rows of Figure 3. One possibility to test the significance on the spatial structure of these eigenvectors is to examine their overlap with another vector that has a clear spatial structure compared with the distribution of overlaps (scalar dot product) of random vectors (Gaussian with zero mean and variance one over the vector length). We find a significant overlap of the 2nd mode (middle column in Figure 3) with the first spatial Fourier mode (planar wave) in the x direction, *p* < 0.01, for each of the presented data sets. However, a stronger indication for the non-randomness of the structure is its stability over different conditions. Comparing Figure 2 and Figure 3 one can see the great similarity in the structure of the eigenvectors—in spite of the facts that (i) Neurons were added (Figure 3 includes also neurons that did not show a clear preferred orientation), (ii) Figure 2 uses stimulus evoked data, whereas Figure 3 uses inter-stimulus-interval data, (iii) The second and third rows in Figure 3 are from a different data set than Figure 2.

We conclude that the correlation matrix reflects several distinct sources: a uniform mode of fluctuation; and modes in which shared fluctuations are stronger among subsets of neurons that are nearby in functional or physical space.

### The effect of collective modes on noise distribution

The eigenvalue of a correlation matrix can be thought of as the variance of the fluctuations in the direction of the corresponding eigenvector. The fluctuations in the responses of different neurons in the direction of a collective mode are correlated and, consequently, will not be averaged out by the summation; rather they will add to yield an eigenvalue that scales linearly with the number of neurons that participate in the mode. Thus, the collective nature of these modes implies that the noise (i.e., the variance or respective eigenvalue of the correlation matrix) in each mode grows linearly with the population size *N*. This is in contrast to shuffled data, in which noise in each mode will remain fixed due to the absence of correlations.

Figure 4 shows the eigenvalue spectrum of the correlation matrix for an example data set, as a function of the population size, *N*. For each value of *N*, we averaged the spectrum over 100 random choices of a population of *N* neurons out of *N*_max_ = 71 in this example (except for populations of 1, 70, and 71 neurons). Thus, the top blue line shows the averaged eigenvalue of the first eigenvector, over 100 realizations of random subpopulation of *N* neurons. Similarly the top green line depicts the average second eigenvalue, and so on. The eigenvectors for smaller populations do not, of course, need to be identical to those identified in the full population; that is, the first eigenvalue may in principle capture a different fluctuation mode for a small population than a large one. However, we found the eigenvectors—particularly the first eigenvector—were remarkably similar across correlation matrices computed from different subpopulations of neurons (see e.g., Figure 2 and first row of Figure 3).

The linear scaling of the eigenvalues of the shared modes implies that for large populations each collective mode accounts for a finite *fraction* of the entire variability. As the total variability in the population (i.e., the normalized variability or trace of the correlation matrix) equals the population size, *N*, this fraction can be estimated by the slope of the linear fit for the scaling of the eigenvalue with the population size. Figure 4B shows the distribution of the fraction of the variance in each of the first three shared modes across the different data sets. The first mode accounts for 11 ± 4%, the second 4 ± 1.4%, and the third 3 ± 1% of the entire variability. Figure 4C shows the eigenvalue spectrum of the correlation matrix as function of the population size for shuffled data for comparison (note the scale). The maximal eigenvalue increases sub-linearly, as expected from the Marcenko-Pasture distribution when the number of trials per eigenvalue is decreased.

It is important to note that correlations do not increase noise but rather re-distribute it. Thus, one can think of the spectrum of the correlation matrix as the distribution of noise in the system. As the sum of all eigenvalues is *N* (population size), the existence of strong shared modes requires the existence of modes with lower (than shuffled) noise levels.

### The effect of collective modes on population codes

The utility of a population code is that, in principle, it allows for the accumulation of signal from many neurons. Shared modes of fluctuation can limit the information content of a population code, as the noise may increase as fast as the signal yielding a finite signal to noise ratio. However, whether noise correlations actually limit information depends also on the distribution of the signal relative to the noise (Averbeck et al., 2006; Kohn et al., 2016). Below we show three examples—applied to both raw and shuffled data—to illustrate how the performance of the decoder can be understood by its relation to the eigenvectors of the correlation matrix.

**Example 1**: The population vector (Georgopoulos et al., 1982), **pv**, is a linear weighted average of the neuronal responses with a specific choice of weights: the response of each neuron is weighted by a two dimensional vector pointing at its preferred orientation, **pv** = **r** · **ff2**. Thus, the population vector can only extract information from the second Fourier mode of the neuronal responses, as a linear projection onto that mode. In most situations, the **pv** is a suboptimal decoder—it extracts less information than that extracted by the best linear decoder. However, this decoder has been widely used in previous work (Georgopoulos et al., 1986; Seung and Sompolinsky, 1993; Salinas and Abbott, 1994; Groh et al., 1997; Sompolinsky et al., 2001; Hohl et al., 2013).

The effect of noise correlations on the population vector is demonstrated in Figure 5. Figure 5A shows the eigenvalue spectrum for this example dataset, which reflects the distribution of noise. The variance of the population vector is determined by the overlap of the population vector weights, $ff2\;=\;(e^{i2\varphi_{1}},e^{i2\varphi_{2}},\ldots e^{i2\varphi_{N}})/\sqrt{N}\;$, with these eigenvectors. Figure 5B shows the (squared) projection of the population vector weights on the different eigenvectors of the correlation matrix, arranged according to their eigenvalue. The population vector has a considerable projection on the several largest shared modes of fluctuation, whereas its projection onto the higher order modes that have less than average variance is rather low. Furthermore, the projection of the population vector onto the shared modes with large eigenvalue converges to a finite limit as *N* grows (Figure 5C). In contrast, the projections onto higher modes, which are added as the population size increases, are decaying fast to zero. The black line shows for comparison the expected overlap of two *N*-dimensional random vectors, which is *1/N*. Thus, the population vector has finite overlap with the collective modes in which the noise grows linearly with *N*. As a result, the total variability of the population vector, which is given by the sum of the product of each eigenvalue (Figure 5A) with its projection onto the population vector (Figure 5B), grows linearly in *N* (Figure 5D). Note that the population vector variability can also be computed directly from the correlation matrix as a bi-linear form (**ff2**)^*t*^**C**(**ff2**). As the signal that the population vector extracts grows linearly with *N* as well, the signal to noise ratio should converge to a finite limit, resulting in the saturation of the population vector accuracy at large *N* (with the asymptotic accuracy depending on the relative rate of increase of the signal and noise).

Figures 5E-H repeat the above exercise with shuffled data. As the amount of data is finite the eigenvalue spectrum and the noise distribution is not completely flat, Figure 5E. However, shuffling removes the structure of the noise resulting in a uniform distribution of signal across all modes, Figures 5F,G. As a result, the total variability of the population vector remains fixed as the population size grows, Figure 5H.

The consequences of the different alignment of the noise with the readout weights, for raw and shuffled data, can be compared directly by looking at population vector accuracy (specifically, the inverse of the population vector angular estimation error variance) in the real and shuffled data (Figure 5I; solid red and dashed magenta lines, respectively). It is important to note that the difference of the population vector accuracy in raw verses shuffled data does not result from the mere fact that largest eigenvalue of the correlation matrix is big for the raw data (c.f. 1st eigenvalue ~8 in the raw data Figure 5A and ~1.4 in shuffled data Figure 5E). But rather from the fact that the largest eigenvalues grow linearly with the population size, Figure 4. Thus, a main distinguishing factor between the raw and shuffled data is the rate in which noise is accumulated by the readout. This can be quantified by the rate of increase of the readout noise with the population size. Specifically, below we shall use the slope of a linear regression to Figures 5D,H.

**Example 2**: Figure 6 presents the signal to noise analysis for the optimal linear estimator (Salinas and Abbott, 1994). For the optimal linear estimator the signal is the vector of co-variation of the response of each neuron with the stimulus (Shamir, 2014). Figure 6B shows how the OLE signal is distributed across the eigenvectors of the correlation matrix, i.e., the (squared) projections of the OLE signal vector on the eigenvectors of the correlation matrix. The signal has a high projection onto the directions with the high eigenvalues; however, it is widely distributed across many directions. Note this is the same data set as in Figure 5. The optimal linear estimator weights take into account both signal (Figure 6B) and noise (Figure 6A for comparison); thus, the optimal linear estimator gives low weights to the two noisiest collective modes, Figure 6C, even though they contain a considerable part of the signal. Consequently, the optimal linear estimator noise is rather similar to the shuffled case in this example, Figure 6D (as in the population vector the noise can be given by **w**^*t*^**Cw**, where **w** is the normalized weight vector of the estimator, note the scale of the ordinate), and its accuracy is also comparable (Figure 6E).

**Figure 8:**
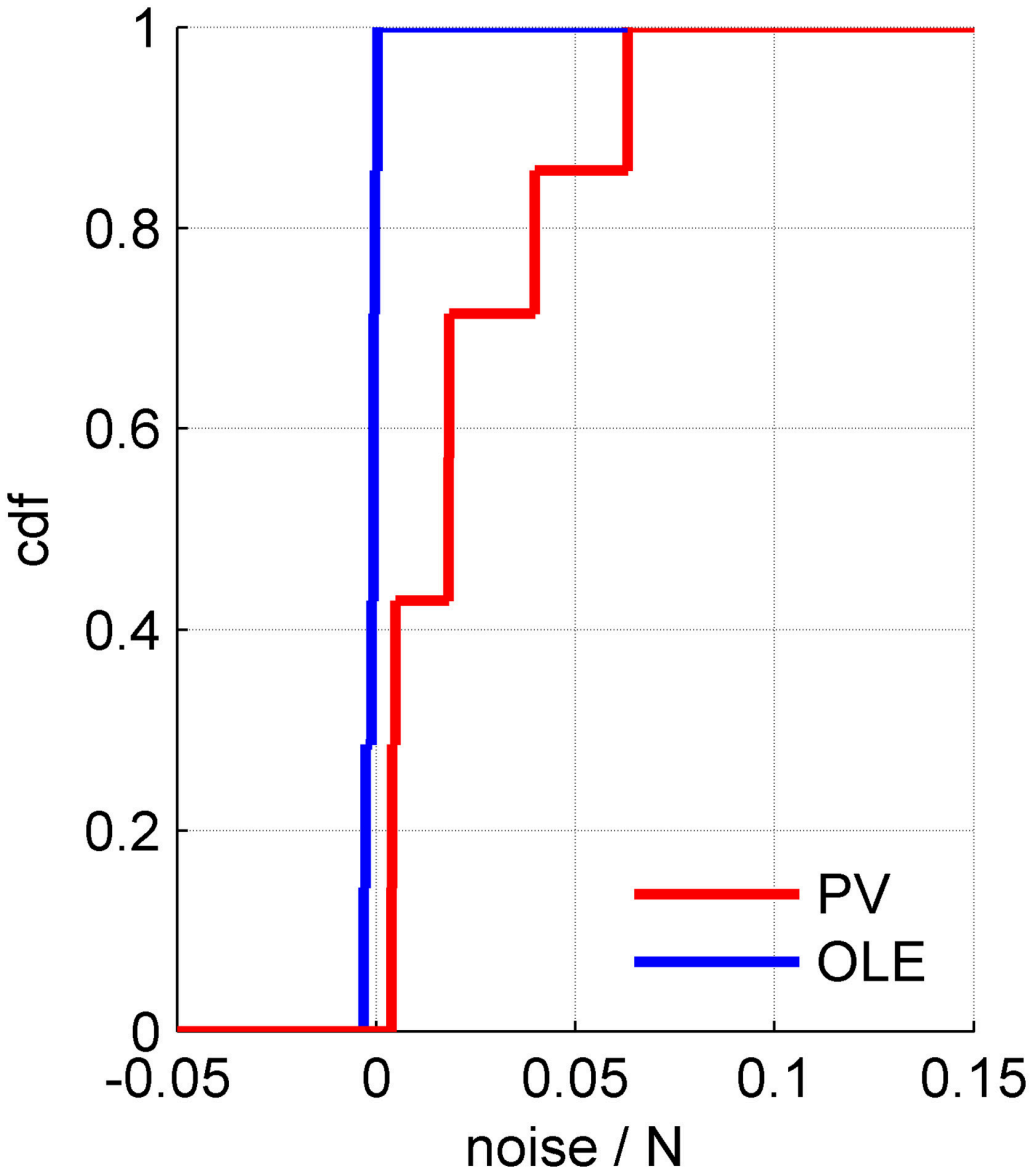
The rate of noise accumulation by different readout algorithms. The cumulative distribution of noise accumulation rate for the population vector (red) and the optimal linear readout (blue) across 7 datasets. The rate was defined as the slope of a linear regression to the increase of the readout noise with the population size, e.g., Figures 5D, 6D, 7D.

**Example 3**: Figure 7 shows a somewhat different scenario, for a different data set. Here, the signal is more widely distributed (compare Figures 6B, 7B). As a result, the optimal linear estimator can give a larger weight to the less noisy modes of the system (compare Figure 6C and Figure 7C). In this case the noise of the optimal linear estimator is reduced relative to the shuffled case, Figure 7D, and its accuracy is higher in the presence of correlations, Figure 7E. Comparing the rate in which the different readouts accumulate noise, we find that for the population vector across datasets this rate of noise accumulation is 0.02 ± 0.02, and always positive, whereas for the optimal linear readout it is −0.0012 ± 0.0013, Figure 8.

### Stability of the global structure of the correlations

The results we have presented above regarding the correlation structure, spectrum and eigenvectors were obtained for averaged correlation matrix. Namely, first the (conditional) covariance matrix of the neural responses was calculated for every given stimulus. Then, the covariance matrix was averaged over the different stimuli, and finally normalized to yield correlation coefficient matrix. Consequently, the results we have shown are for “stimulus independent” correlations, as we averaged over all stimulus conditions.

**Figure 9:**
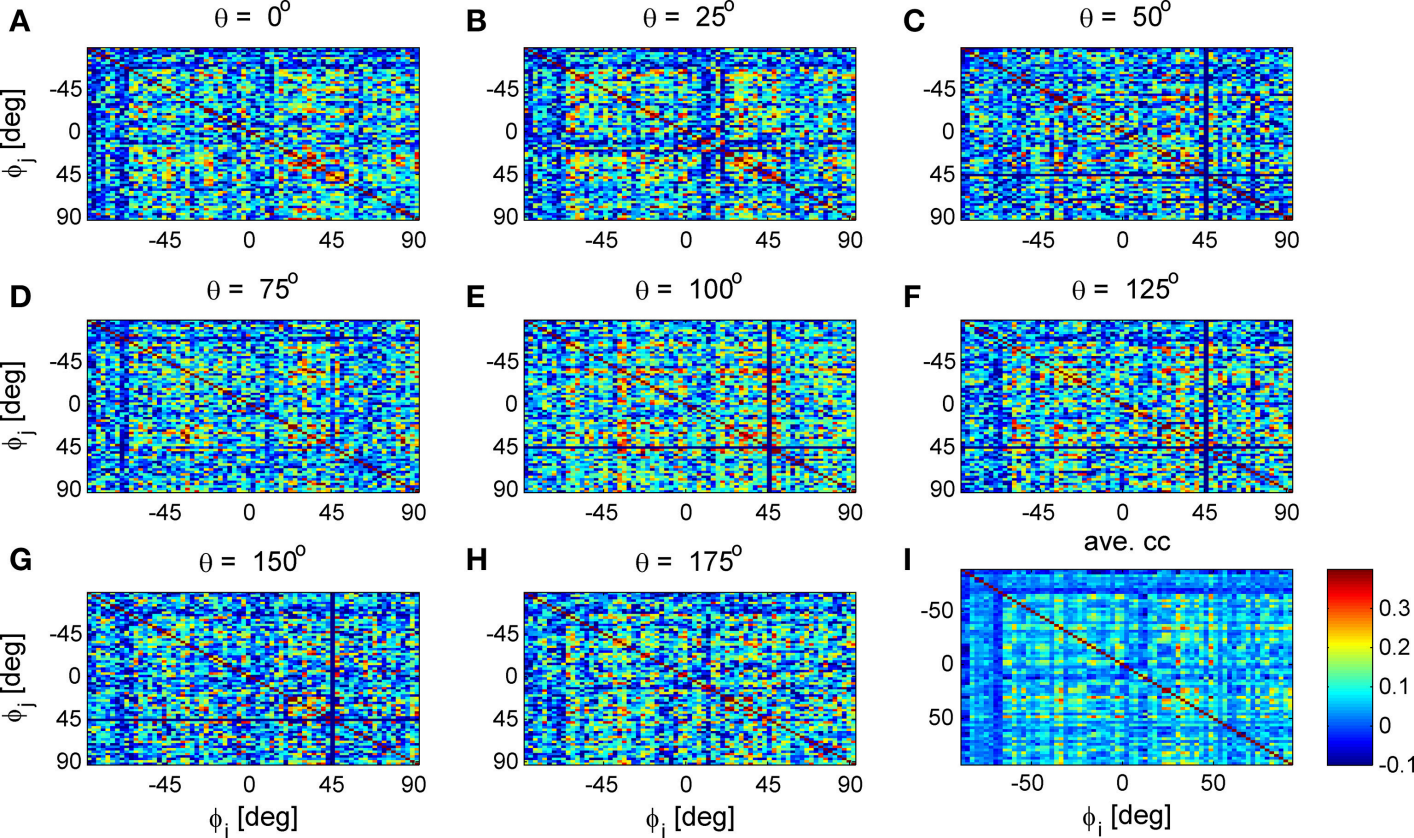
Stimulus dependent correlation matrices. Nine correlation matrices from the same data set are shown in color code as function of the neuron's preferred orientation: **(A-H)** Show the correlation matrices for a given stimulus orientation (out of 36 stimuli). **(I)** Shows the mean correlation matrix.

Figure 9 shows 8 correlation matrices for one data set, each for a different stimulus orientation (out of 36 stimuli). The mean correlation matrix is shown at the bottom right. The estimated correlation matrices are similar to each other but not identical. However, this is to be expected as each stimulus-dependent correlation matrix has *N*(*N* − 1)/2 ≈ 2500 different elements and only *T* = 135 trials per stimulus to estimate them. One would expect fluctuations with standard deviation of $1/\sqrt{T}\approx$0.086 (if there are no correlations). Consequently, the main noticeable difference is that the (finite sample estimation) noise in the averaged (or “stimulus independent”) correlation matrix is smaller by a factor of $\sqrt{36}=6$ (see also [32]).

**Figure 10:**
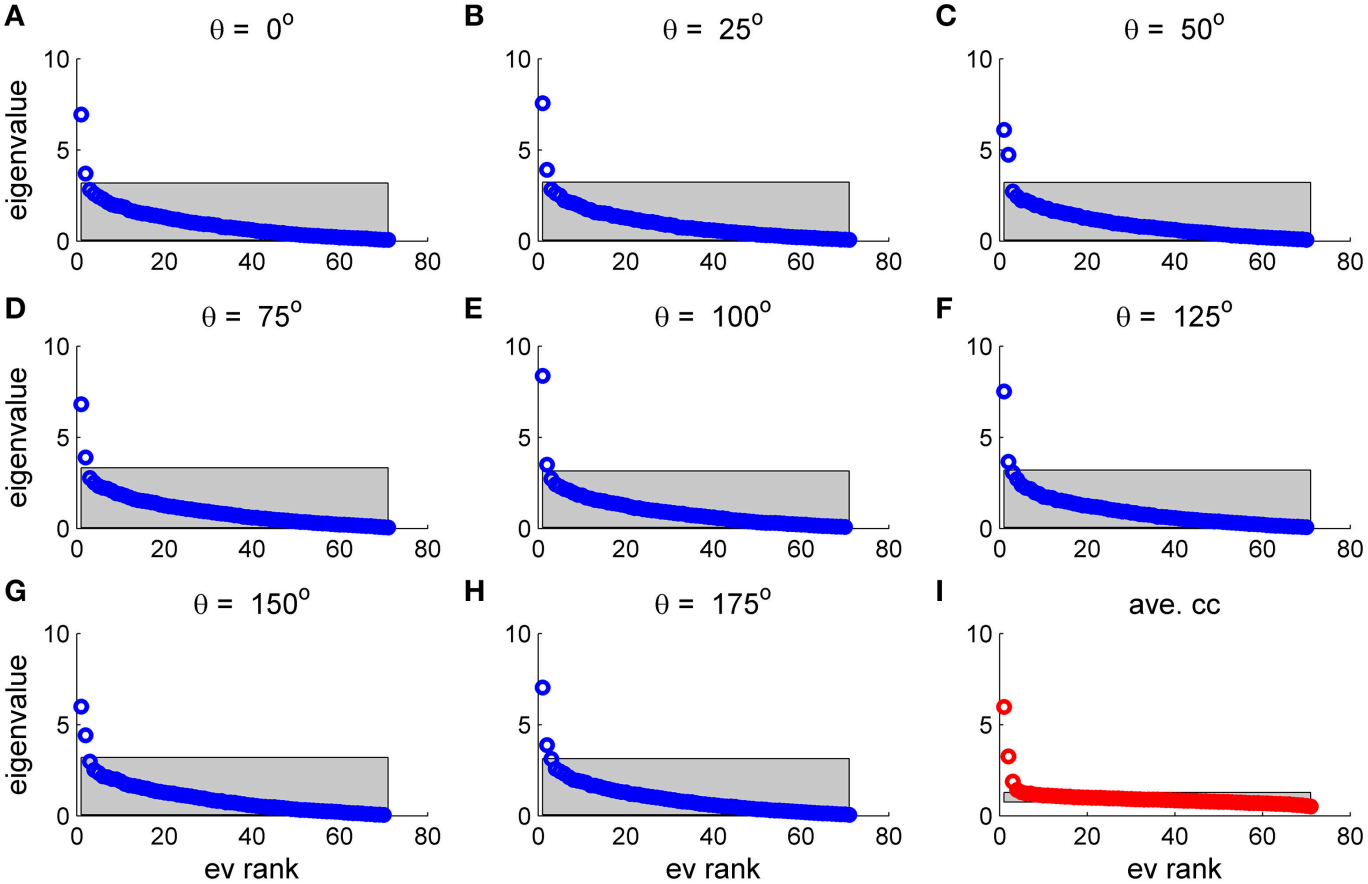
Eigenvalue distribution for stimulus dependent correlation. The distribution of eigenvalues for the 9 matrices in this figure is shown by rank order (open circles). The gray region depicts the range between the maximal eigenvalue obtained over 1,000 realizations of shuffled data and the minimal one for each case. **(A–H)** Show the distribution for a given stimulus orientation (out of 36 stimuli). **(I)** Shows the distribution for the mean correlation matrix.

**Figure 11:**
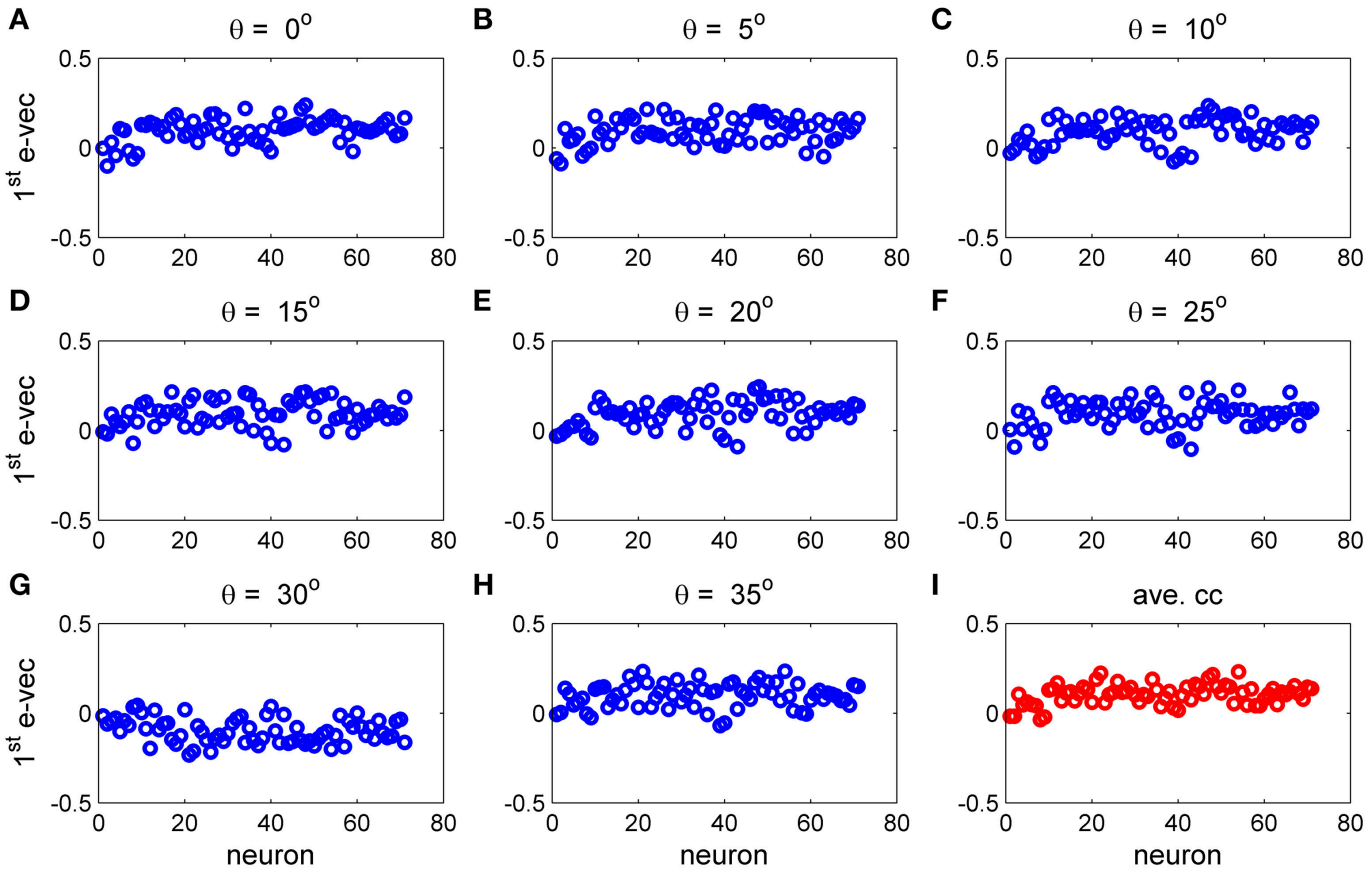
The principal eigenvector for stimulus dependent correlation. The eigenvector corresponding to the largest eigenvalue for the 9 matrices in Figure 10 and this figure is shown by neuron number. **(A–H)** Show the principal eigenvector for a given stimulus orientation (out of 36 stimuli). **(I)** Shows the principal eigenvector for the mean correlation matrix.

To study the similarity of the correlation matrices we examined more global features. Figures 10,11 show the spectra and first eigenvector for the eight conditional (or “stimulus dependent”) correlation matrices in blue and of the average correlation matrix in red (bottom right). The eigenvalue spectrum shows a distinct qualitative similarity: two relatively well separated principle eigenvalues followed by a semi-continuous tail of smaller eigenvalues. The different eigenvectors also seem to share qualitatively similar features. To quantify their similarity we computed the cosine of the angle between each eigenvector for a given stimulus and the corresponding eigenvector of the averaged correlation matrix. We find that for the first eigenvalue the cosine is 0.93 ± 0.02 (mean± std across the 36 different orientations) and for the second eigenvector 0.83 ± 0.05. Across datasets the mean cosine for the first eigenvector is 0.88 ± 0.12, for the second eigenvector 0.63 ± 0.21, and for typical eigenvector 0.15 ± 0.08. In addition, we find similar correlation structure during the inter-stimulus-interval. For the specific example of Figure 11 the cosine between the first eigenvector of the average correlation matrix and the inter-stimulus-interval is 0.8 and for the second eigenvector 0.6. Across datasets the cosine between of the angle the first eigenvector of the average correlation matrix and of the inter-stimulus-interval correlation matrix is 0.87 ± 0.1, and for the second eigenvector 0.57 ± 0.32.

## Discussion

We find that neuronal noise correlations have a non-trivial structure. Trial-to-trial fluctuations are composed of a few large collective modes of fluctuations and a semi-continuous tail of small eigenvalues (Figure 1D). The magnitude of the fluctuations in the collective modes grows linearly with the population size with a ratio that decreases with the order of the mode (Figure 4B). Consequently, as the population size grows, more eigenvectors become significant (Figure 4A). We find that about 10% of the eigenvalues are collective modes (Figure 1D, inset).

The structure of the collective modes is extended, involving most of the neurons in the population (Figure 2). The largest collective mode represents a “uniform” mode of fluctuations, as observed in both awake (Arieli et al., 1996; Yu et al., 2011; Rabinowitz et al., 2015; Engel et al., 2016) and anesthetized (Ecker et al., 2014; Lin et al., 2015; Schölvinck et al., 2015) animals. The second and third modes often have spatial or functional structure. It is important to note that the ability to detect additional structure requires sufficient data, both sufficient trial number to accurately estimate the correlation matrix and sufficient neurons to see more distributed patterns of activity. It is easy to miss non-uniform modes with less data (see e.g., Figure 10).

**Figure 12:**
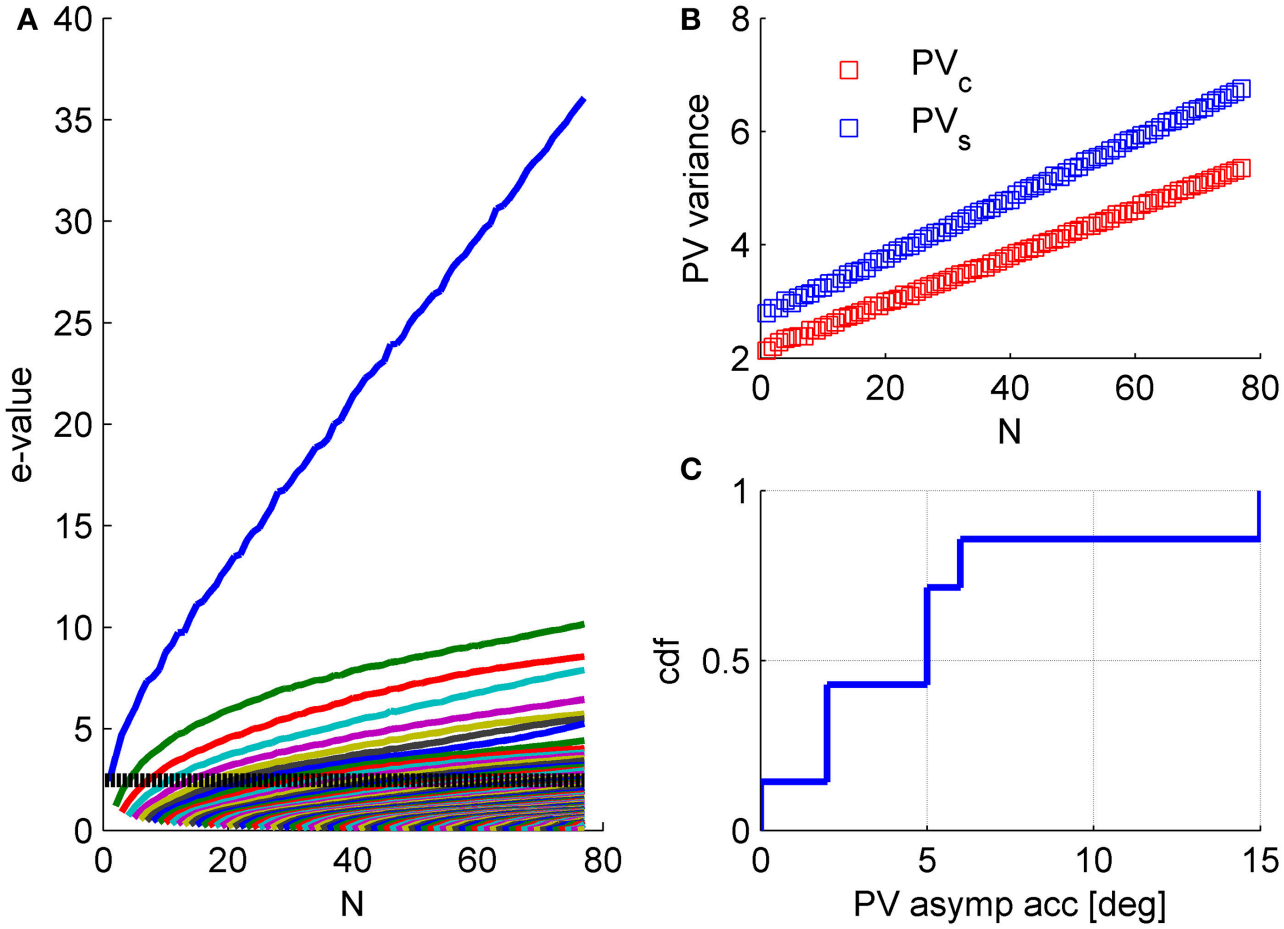
Structure of the covariance matrix. **(A)** The eigenvalue spectrum of the response covariance matrix is shown as function of the population size (same data set as in Figures 1, 5, 6). For every population size, *n*, the eigenvalue spectrum was averaged over 1,000 (compare with 100 for the correlation matrix spectrum in Figure 4) random choices of subpopulations of n neurons out of *N* = 77 (except for *n* = 1, *N*−1, and *N*). The dashed line shows the mean variance in the population. **(B)** The population vector noise is plotted as function of the population size. We separated the noise into two contributions. The first contribution is to fluctuations in the magnitude of the population vector, i.e., projection on **PV**_*c*_$=\left(\cos2\varphi_{1},\cos2\varphi_{2},\ldots \cos2\varphi_{N}\right)\sqrt{2/N}$ (blue), where we measure the preferred orientations of the neurons relative to stimulus orientation. These fluctuations do not affect the angular estimation of the population vector (that we focused on here). However, one may consider scenarios in which the magnitude of the population vector carries relevant information e.g., stimulus contrast or degree of confidence in the estimation. Thus, fluctuations in the magnitude of the population vector may affect the estimation of contrast or the confidence in the angular estimate, but will not affect the angular estimation of the orientation. The second, contribution is to fluctuations in the tangential direction of the population vector, i.e., projection on **PV**_*s*_$=\left(\sin2\varphi_{1},\sin2\varphi_{2},\ldots \sin2\varphi_{N}\right)\sqrt{2/N}$ (blue). Fluctuations in this direction will affect the angular estimation of the orientation. **(C)** Assuming the variability in the direction of **PV**_*s*_ will continue to scale linearly with the population size one can compute the asymptotic accuracy of the population vector as (square root of) the ratio of linear slopes of noise and signal (where the signal is the projection of the population tuning curve on the direction of **PV**_*c*_) in the limit of large N. Note that the difference in the rate of noise growth in the radial and tangential directions (slope of blue vs. red curves in **B**) reflects stimulus dependence of the correlations.

Our analysis of the structure of neuronal noise correlations was based on studying the correlation coefficients matrix, as is customary in the field. However, when studying the accuracy of linear population codes the covariance matrix is the more natural quantity. The use of correlation coefficient matrix facilitates the comparison with other studies. In addition, it assists the investigation of the underlying structure by removing the considerable variability on the diagonal of the covariance matrix, namely the distribution of variances. Nevertheless, qualitatively similar findings arise from the investigation of the covariance matrix, Figure 12.

The magnitude of the collective modes is relatively small. The relative strength of the mode, in terms of the eigenvalue divided by *N*, is typically about 10% for the “uniform mode” and less than 5% for other modes. However, their potential effect on information coding results not from their relative size, but from the fact that their variance grows with the population size, causing decoding accuracy to asymptote to a finite value as population size increases, at least for the population vector, Figure 12C. Thus, we emphasize, that these results are fundamentally different than the common example of how the accuracy of a suboptimal readout is less than that of the optimal. The difference in the population vector and the optimal linear readout performances is not quantitative, but qualitative. The saturation of the population vector accuracy is evident from the linear scaling of its noise (Figure 12B), which allows us to compute its asymptotic accuracy (Figure 12C).

On the other hand, the performance of the optimal linear estimator does not appear to show any sign of correlations limiting information–at least for populations of about 100 neurons. The main difference for the different behavior of the population vector and the optimal linear readout is that the population vector defines its weights based only on the signal, whereas the optimal linear readout takes into account the noise distribution as well. Why do we fail to observe saturating performance, as predicted by finite psychophysical accuracy (Moreno-Bote et al., 2014)? Recent work estimated the required number of simultaneously recorded neurons to observe the saturating effect to be on the order of several thousands (Kanitscheider et al., 2015), whereas in our datasets we have less than 100 neurons. As we find that about 10% of the modes are collective modes of fluctuations, it is reasonable to assume that for a population of several thousands of neurons additional modes will manifest possibly including information limiting modes. Note, however, that such population size may also require collecting an order of magnitude more trials in order to obtain a fair estimate of the correlation matrix.

In some cases the accuracy of the optimal linear readout is better in the presence of correlations than in shuffled data. This finding has been presented recently in some studies as beneficial correlations (Lin et al., 2015; Franke et al., 2016; Zylberberg et al., 2016). Alternatively, one may argue that the comparison with shuffled data is unclear. Shuffling does remove correlated noise, rather it re-distributes it (Shamir, 2014).

Recently, it was suggested that stimulus dependent correlations improve coding accuracy of linear readouts and that stimulus independent correlations that are obtained by averaging the correlations over different stimulus conditions are harmful. Could this be the reason for the information limiting effect of the population vector accuracy? No – the error of the population vector estimation was not computed on averaged data. Care was also taken to separate the data to training set and generalization set. The training set was used to define the preferred orientations of the neurons for the population vector. The generalization set was used to estimate the error. Furthermore, we find high similarity in the global features of the noise structure between the stimulus dependent correlation matrices, the average stimulus independent matrix and the inter-stimulus interval correlation structure. Nevertheless, this should not be taken to imply that the correlations are independent of the stimulus or that this dependence has no computational implications. The difference in the rate of noise growth in the radial and tangential directions of the population vector (slope of blue vs. red curves in Figure 12B) reflects stimulus dependence of the correlations.

Ecker et al. (2014) used Gaussian process factor analysis to investigate the correlations structure. They find that most of the correlated variability can be accounted for by a single global parameter that modulates the activity of the entire population, which they link to the “brain state transitions under anesthesia.” Schölvinck et al. (2015) reported that a single uniform mode of fluctuations accounts for most of the correlated variability in their measured responses. Similarly, we also find that the most prominent collective mode of fluctuations is the uniform mode. It is important to note that such common modes are likely prominent in the awake state (Arieli et al., 1996; Yu et al., 2011; Rabinowitz et al., 2015; Engel et al., 2016), although they may be enhanced under anesthesia. However, in addition to the uniform mode we find additional collective modes of fluctuation that exhibit a clear meaning. Although these additional modes are considerably weaker than the uniform mode their effect on population codes may be dramatic and highly dependent upon the readout algorithm used to extract the information from the neuronal population response.

## Ethics statement

This work focuses on data analysis. All data analyze here is courtesy of the laboratory of Adam Kohn. All procedures were approved by the Institutional Animal Care and Use Committee at the Albert Einstein College of Medicine of Yeshiva University, and were in compliance with the guideline set forth in the United States Public Health Service Guide for the Care and Use of Laboratory Animals.

## Author contributions

MS conceived and designed research; OM and MS analyzed data. Both authors reviewed the last version of the manuscript.

### Conflict of interest statement

The authors declare that the research was conducted in the absence of any commercial or financial relationships that could be construed as a potential conflict of interest.
